# Broadly neutralizing human monoclonal antibodies to the hepatitis C virus E2 glycoprotein

**DOI:** 10.1099/vir.0.83386-0

**Published:** 2008-03

**Authors:** Ania M. Owsianka, Alexander W. Tarr, Zhen-Yong Keck, Ta-Kai Li, Jeroen Witteveldt, Richard Adair, Steven K. H. Foung, Jonathan K. Ball, Arvind H. Patel

**Affiliations:** 1 MRC Virology Unit, Institute of Virology, University of Glasgow, Church Street, Glasgow G11 5JR, UK; 2 The Institute of Infection, Immunity and Inflammation, School of Molecular Medical Sciences, The University of Nottingham, Queen's Medical Centre, Nottingham NG7 2UH, UK; 3 Department of Pathology, Stanford University School of Medicine, Stanford, CA 94305, USA

## Abstract

The humoral response to hepatitis C virus (HCV) may contribute to controlling infection. We previously isolated human monoclonal antibodies to conformational epitopes on the HCV E2 glycoprotein. Here, we report on their ability to inhibit infection by retroviral pseudoparticles incorporating a panel of full-length E1E2 clones representing the full spectrum of genotypes 1–6. We identified one antibody, CBH-5, that was capable of neutralizing every genotype tested. It also potently inhibited chimeric cell culture-infectious HCV, which had genotype 2b envelope proteins in a genotype 2a (JFH-1) background. Analysis using a panel of alanine-substitution mutants of HCV E2 revealed that the epitope of CBH-5 includes amino acid residues that are required for binding of E2 to CD81, a cellular receptor essential for virus entry. This suggests that CBH-5 inhibits HCV infection by competing directly with CD81 for a binding site on E2.

Hepatitis C virus (HCV) is a small, enveloped, positive-strand RNA virus that infects an estimated 170 million individuals worldwide (Anonymous, 1999). The virus evades the immune system so successfully that most of those who contract it remain chronically infected and may eventually develop serious liver disease. HCV disables the innate antiviral response by blocking pathways of interferon induction and signalling (Gale & Foy, 2005; Meylan & Tschopp, 2006) and escapes the adaptive immune response largely by means of its genetic variability (Bowen & Walker, 2005; Brown *et al.*, 2005; Farci *et al.*, 2000; Shimizu *et al.*, 1994; Thimme *et al.*, 2006). There are six distinct genotypes of HCV, each divided into subtypes (Simmonds *et al.*, 2005), and within a single individual the virus exists as a constantly evolving quasispecies (Bukh *et al.*, 1995).

An effective T-cell response is essential for controlling HCV infection (Bowen & Walker, 2005; Shoukry *et al.*, 2004), but neutralizing antibodies also play an important role. It was observed long ago that immunoglobulin prophylaxis protected against HCV infection (Conrad & Lemon, 1987; Knodell *et al.*, 1976) and it has recently been shown that this protective effect correlates with the presence of antibodies that can neutralize retroviral pseudoparticles bearing HCV envelope glycoproteins (HCVpp) (Yu *et al.*, 2004). Studies of acutely infected cohorts show that rapid induction of neutralizing antibodies may afford protection (Lavillette *et al.*, 2005a; Pestka *et al.*, 2007). Moreover, antibodies present during the acute phase of infection can neutralize an infectious dose of HCV administered to chimpanzees (Farci *et al.*, 1994).

The envelope glycoproteins E1 and E2 are the natural targets for neutralizing antibodies (Bartosch *et al.*, 2003a; Rosa *et al.*, 1996). E2 binding to CD81 on the cell surface is necessary, but not sufficient, for infection (Cocquerel *et al.*, 2006; Kapadia *et al.*, 2007; Koutsoudakis *et al.*, 2007). The first hypervariable region (HVR-1) within the amino-terminal part of E2 is involved in virus binding and entry (Bartosch *et al.*, 2003c) and is immunodominant. Antibodies to HVR-1 neutralize infection, but they are typically isolate-specific, with little or no recognition of other isolates or genotypes. In contrast, conformational epitopes on E2 outside HVR-1 are less prone to variation and are able to elicit more broadly neutralizing antibodies (Habersetzer *et al.*, 1998; Hadlock *et al.*, 2000; Johansson *et al.*, 2007; Perotti *et al.*, 2008; Schofield *et al.*, 2005).

We previously reported the generation of a panel of IgG_1_ human monoclonal antibodies (HmAbs) from the peripheral B cells of an individual with asymptomatic HCV genotype 1b infection (Hadlock *et al.*, 2000). They recognize conformational epitopes on E2 of more than one genotype, and have been mapped to distinct domains, designated A, B and C (Keck *et al.*, 2004). The HmAbs that recognize domains B and C block E2 binding to CD81 and neutralize genotype 1a HCVpp and genotype 2a JFH-1 cell culture-infectious HCV virions (HCVcc), whereas HmAbs to domain A do not block CD81 binding and are non-neutralizing (Hadlock *et al.*, 2000; Keck *et al.*, 2004, 2005, 2007; Op De Beeck *et al.*, 2004).

Recent success in growing the genotype 2a JFH-1 isolate of HCV in cultured cells to produce infectious virions (Lindenbach *et al.*, 2005; Wakita *et al.*, 2005; Zhong *et al.*, 2005) has yet to be replicated for all of the other genotypes. However, various aspects of HCV binding and entry, including antibody neutralization, can be studied by using HCVpp (Bartosch *et al.*, 2003b; Hsu *et al.*, 2003). We have assembled a panel of functional E1E2 glycoprotein sequences representing all major genotypes (Lavillette *et al.*, 2005b; Owsianka *et al.*, 2005; Tarr *et al.*, 2006), allowing us to extend the characterization of our previously described HmAbs.

HCVpp displaying E1E2 of genotypes 1–6 and carrying a luciferase reporter gene were generated in HEK293T cells and purified as described previously (Bartosch *et al.*, 2003b; Owsianka *et al.*, 2005). Aliquots of the HCVpp were mixed with 100 μg ml^−1^ of each HmAb, incubated for 1 h at 37 °C and used to infect Huh-7 cells (Nakabayashi *et al.*, 1982). Luciferase activity in the infected cells was measured after 4 days.

Domain B (CBH-2, -5, -8C and -11) and domain C (CBH-7) HmAbs each had a distinct spectrum of neutralizing activity against the genotype panel (Fig. 1a[Fig f1]), whereas domain A HmAbs did not reduce the infectivity of any genotype by more than 25 % (not shown). An isotype-matched control HmAb against HCMV p64 (R04) did not reduce the infectivity of any genotype by more than 10 % (Fig. 1a[Fig f1]).

CBH-5 reduced infectivity of HCVpp representing all genotypes by at least 40 %, whilst the other domain B HmAbs each inhibited a more restricted range of genotypes. CBH-2 neutralized HCVpp of genotypes 2a, 5 and 6 by at least 70 %, and genotypes 2b and 4 by just under 50 %. It did not neutralize genotype 3a or genotype 1a clone H77c, although it neutralized the closely related clone H by almost 50 % (Fig. 1). These two clones differ at five amino acid positions within E2, which may account for the difference in CBH-2 neutralization. CBH-8C and CBH-11 did not neutralize either of the 1a isolates, consistent with previous findings that these HmAbs do not recognize E2 of genotype 1a strain H (Keck *et al.*, 2004). CBH-8C neutralized genotypes 1b, 2a, 2b, 3a and 6, whilst CBH-11 showed modest neutralization of genotypes 1b, 2a, 2b and 6. The domain C HmAb CBH-7 neutralized both the 1a strain H clones by more than 70 %, genotypes 1b, 2a and 2b by about 40 % and genotype 4 by 20 %, but had no effect on genotypes 3a, 5 or 6.

To examine the correlation between binding and neutralization, we tested the binding of antibodies CBH-2, -5 and -7 to E2 displayed on HCVpp. After sucrose-density equilibrium centrifugation in 20–60 % sucrose at 270 000 ***g*** for 18 h at 4 °C, the infectious gradient fractions were pooled, captured onto GNA (*Galanthus nivalis* agglutinin)-coated plates and probed with each HmAb over a range of concentrations. Bound antibodies were detected with alkaline phosphatase-conjugated goat anti-human IgG followed by *p*-nitrophenylphosphate disodium hexahydrate, and *A*_405_ was measured.

CBH-5 was the most broadly reactive HmAb, giving a robust, concentration-dependent signal with each genotype (Fig. 1b[Fig f1]). This broad reactivity clearly underpins its ability to neutralize HCVpp across the spectrum of genotypes. CBH-2 gave a concentration-dependent signal with E2 of all genotypes except 1a H77c and 3a (Fig. 1b[Fig f1]), which agrees with its lack of ability to neutralize HCVpp bearing these two sequences. Binding to genotype 4 was detectable only at high CBH-2 concentrations. CBH-7 gave a strong concentration-dependent signal with genotypes 1 and 2, and a weaker one with genotypes 3–6, in agreement with its neutralization profile. However, the relationship between binding and neutralization was more complex for CBH-7, as it recognized genotype 6 E2, but did not neutralize genotype 6 HCVpp. It may be that greater saturation with CBH-7 is required for neutralization or that there is a difference in the mechanism of neutralization between CBH-7 and the domain B antibodies.

We showed previously that CBH-5 and the other domain B HmAbs potently neutralize genotype 2a (JFH-1) HCVcc, whilst CBH-7 has modest HCVcc-neutralizing activity (Keck *et al.*, 2007). The half-maximal inhibitory concentration (IC_50_) of these HmAbs is considerably lower for HCVcc than for HCVpp (Keck *et al.*, 2007). To see whether this holds true for other isolates, we generated an intragenotypic JFH-1 chimera carrying genotype 2b (UKN2B1.1; Owsianka *et al.*, 2005) E1E2 glycoproteins. The 2b HCVcc were pre-incubated with HmAbs CBH-2, -5 and -7 over a range of concentrations before infecting Huh-7 cells. After 4 days, the levels of viral RNA in these cells were determined by real-time PCR (qRT-PCR) using relative quantification, where each sample was normalized to an endogenous control gene (glyceraldehyde-3-phosphate dehydrogenase).

All three HmAbs were very effective at reducing infectivity (Fig. 2a[Fig f2]), and the order of neutralization potency of these antibodies for the 2b chimeric virus was the same as for the genotype 2a virus: CBH-5>CBH-2>CBH-7. A similar titration carried out with HCVpp displaying UKN2B1.1 E1E2 glycoproteins showed that all three HmAbs inhibited infection at concentrations approximately two orders of magnitude higher than those required for HCVcc neutralization (Fig. 2b, c[Fig f2]).

The difference between the IC_50_ (and IC_90_) values of all three antibodies for HCVcc and HCVpp is striking. This could be related to altered exposure or accessibility of antibody epitopes, possibly resulting from differences in glycosylation of E2 in the two systems, the lack of NS2 in HCVpp or the size difference between HCVpp and authentic HCV virions (Sandrin *et al.*, 2005). However, the simplest explanation is that there is more non-infectious E2 relative to infectious particles in a preparation of HCVpp than of HCVcc, and so more antibody is required to neutralize HCVpp. Ideally, one would compare the neutralization of equal numbers of particles or of equal amounts of E2 presented on the two types of particles, but it would be very hard to achieve this with any degree of accuracy. The HCVpp system has been used in several studies to determine virus neutralization by patient sera (Farci *et al.*, 1994; Lavillette *et al.*, 2005a; Pestka *et al.*, 2007; Yu *et al.*, 2004). Our results indicate that modest inhibition of HCVpp may translate into more substantial neutralization of infectious virus.

Domain B and C HmAbs all have neutralization-of-binding (NOB) activity (Hadlock *et al.*, 2000), so it is likely that their epitopes overlap with regions of E2 involved in CD81 binding. Antibody blocking experiments have implicated several regions of E2 in CD81 binding (Clayton *et al.*, 2002; Flint *et al.*, 1999; Owsianka *et al.*, 2001). We identified conserved amino acid residues within these regions and, using the 1a H77c sequence as a wild type, made a panel of mutants in which each conserved residue in turn was replaced by alanine. Some additional mutants, available from other studies, were also included. The mutations did not affect the overall conformation of the E2 protein or its ability to form non-covalent heterodimers with E1 (Owsianka *et al.*, 2006). The mutant proteins were expressed transiently in HEK 293T cells; equivalent amounts of monomeric E2 protein were captured on GNA plates and probed with antibodies CBH-5 and CBH-7 at 2 μg ml^−1^. CBH-5 binding was abrogated completely by mutations G523A, P525A, G530A, D535A and N540A, indicating that these amino acid residues may be involved in its recognition of E2 (Fig. 3). Residues G523, P525, G530 and D535 are completely conserved in functional sequences across all genotypes, whereas N540 is not (Owsianka *et al.*, 2006). G530 and D535 are essential for CD81 interaction with E2, and G523 is also likely to be involved (Owsianka *et al.*, 2006). The residue corresponding to D535 in the JFH-1 virus is essential for infectivity, as a point mutation at this position renders HCVcc non-infectious (J. Witteveldt, unpublished results). These data indicate that the epitope of CBH-5 includes four highly conserved amino acid residues, three of which are involved in CD81 binding, suggesting that CBH-5 exerts its potent neutralization of HCV infectivity by competing directly with CD81 for binding to E2. This is likely to account for its broad spectrum of activity, as any changes in the CBH-5 epitope would be likely to affect CD81 binding and virus entry.

N540 is important for recognition by both antibodies because it is a glycosylation site, mutation of which probably induces a local conformational change in E2, which does not impair E2 folding and function severely, but reduces the affinity of various neutralizing antibodies including CBH-5 and CBH-7 (Goffard *et al.*, 2005; Helle *et al.*, 2007). The only other mutation that reduced the binding of CBH-7 substantially was W549A (Fig. 3). W549 is a highly conserved residue that is essential for HCVpp infectivity, but not for CD81 binding (Owsianka *et al.*, 2006), which suggests that CBH-7 may act by steric hindrance rather than by direct competition with CD81. Our panel of E2 proteins did not include mutants in all regions of E2 that have been implicated in CD81 binding; other studies have demonstrated the involvement of residues 436–443 (Drummer *et al.*, 2006) and 613–618 (Roccasecca *et al.*, 2003). Further work is in progress to define the epitopes of the domain B and C HmAbs more completely, and how these relate to regions involved in CD81 binding. It was not possible to use the same panel of mutant E2 proteins to map the epitopes of CBH-2, -8C and -11, as these bind poorly to the wild-type H77c sequence.

In this study of five conformation-sensitive anti-E2 HmAbs, tested for their ability to neutralize HCVpp pseudotyped with glycoproteins of HCV genotypes 1–6, only one antibody, CBH-5, was found to be active against every E2 sequence. This highlights the need to use E1E2 from the full range of genotypes to identify truly broadly neutralizing antibodies. Cross-reactive HmAbs specific for conformational epitopes on E2 have been isolated and characterized in several laboratories (Allander *et al.*, 2000; Bugli *et al.*, 2001; Eren *et al.*, 2006; Perotti *et al.*, 2008; Schofield *et al.*, 2005), but such broad reactivity across all genotypes has rarely been found (Johansson *et al.*, 2007).

Broadly neutralizing antibodies are potentially of direct clinical relevance. They could be used prophylactically to reduce the risk of HCV infection after needlestick or other accidental exposure. In the liver-transplant setting, it would be very desirable to reduce the incidence of graft reinfection by passive immunotherapy with antibodies such as CBH-5. The characteristics of CBH-5 suggest that there is at least one highly conserved neutralizing epitope on the E2 glycoprotein to which the human immune system is capable of mounting a response. This is encouraging for future vaccine development, although focusing the immune response on this epitope will be challenging, given its conformational nature. Greater knowledge of the structure of the HCV glycoproteins, coupled with detailed mapping of the epitopes of other neutralizing antibodies, should bring us closer to this goal.

## Figures and Tables

**Fig. 1. f1:**
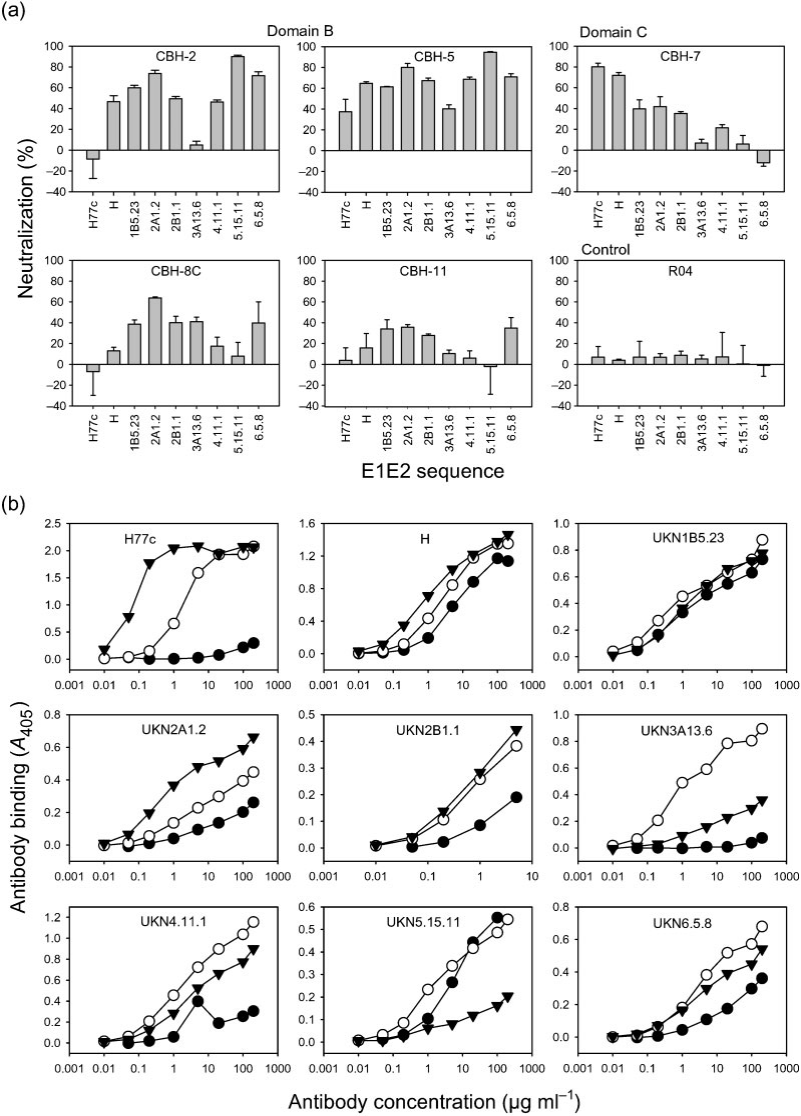
(a) Neutralization by HmAbs of HCVpp pseudotyped with E1E2 sequences of genotypes 1–6. This dataset is representative of results obtained in at least three independent experiments. (b) Reactivity of HmAbs CBH-2 (•), CBH-5 (○) and CBH-7 (▾) with purified HCVpp in GNA ELISA. The following E1E2 sequences were used: genotype 1a, H77c (GenBank accession no. AF011751) and H (Bartosch *et al*., 2003b; Helle *et al*., 2007); genotype 1b, UKN1B5.23 (AY734976); genotype 2a, UKN2A1.2 (AY734977); genotype 2b, UKN 2B1.1 (AY734982); genotype 3a, UKN3A13.6 (AY894683); genotype 4, UKN4.11.1 (AY734986); genotype 5, UKN5.15.11 (AY894682); genotype 6, UKN6.5.8 (EF427671).

**Fig. 2. f2:**
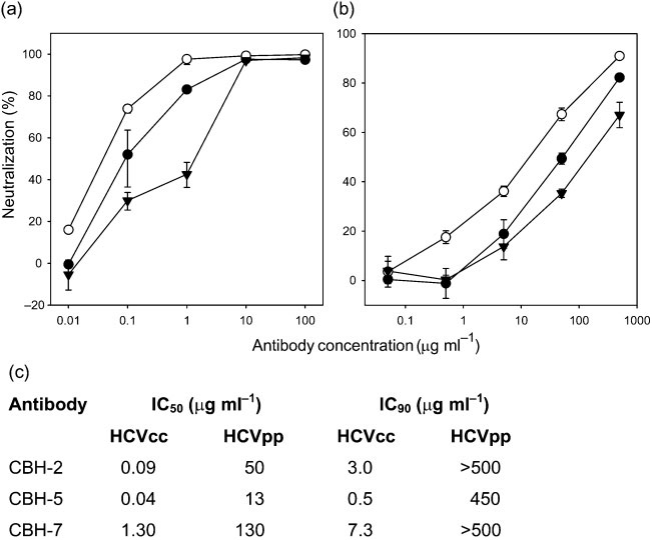
Neutralization by HmAbs CBH-2 (•), CBH-5 (○) and CBH-7 (▾) of (a) genotype 2b HCVcc and (b) genotype 2b HCVpp. (c) IC_50_ and IC_90_ of each HmAb for genotype 2b HCVcc and HCVpp.

**Fig. 3. f3:**
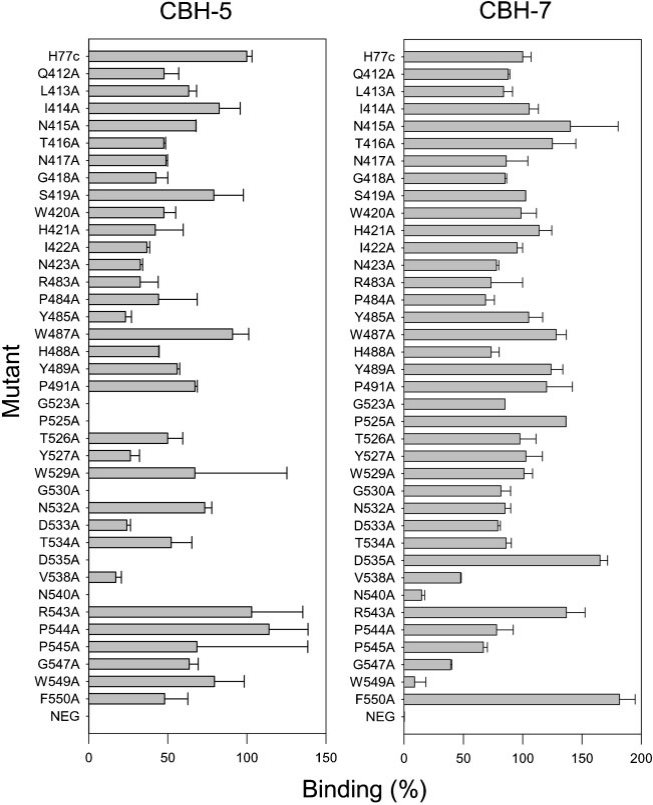
Alanine-replacement mutagenesis. Mutant proteins are annotated according to the amino acid in the H77c sequence, the amino acid position relative to the start of the H77 polyprotein chain and the substitution introduced. Binding of antibody to each mutant protein is expressed as a percentage of binding to wild-type H77c protein.
